# The Complex Role of Matrix Metalloproteinase-2 (MMP-2) in Health and Disease

**DOI:** 10.3390/ijms252413691

**Published:** 2024-12-21

**Authors:** Marta Wolosowicz, Slawomir Prokopiuk, Tomasz W. Kaminski

**Affiliations:** 1 Department of Pharmacology & Chemical Biology, University of Pittsburgh, Pittsburgh, PA 15260, USA; 2 Faculty of Health Sciences, University of Lomza, 14 Akademicka St., 18-400 Łomża, Poland; sprokopiuk@al.edu.pl; 3 Pittsburgh Heart, Lung and Blood Vascular Medicine Institute (VMI), University of Pittsburgh School of Medicine, Pittsburgh, PA 15260, USA; 4 Thrombosis and Hemostasis Program, VERSITI Blood Research Institute, Milwaukee, WI 53226, USA

**Keywords:** matrix metalloproteinase-2, MMP-2, extracellular matrix, ECM, tissue remodeling

## Abstract

Matrix metalloproteinase-2 (MMP-2), a zinc-dependent enzyme, plays a critical role in the degradation and remodeling of the extracellular matrix (ECM). As a member of the gelatinase subgroup of matrix metalloproteinases, MMP-2 is involved in a variety of physiological processes, including tissue repair, wound healing, angiogenesis, and embryogenesis. It is primarily responsible for the degradation of type IV and V collagen, fibronectin, laminin, and elastin, which are essential components of the ECM. MMP-2 is secreted as an inactive pro-enzyme (proMMP-2) and activated through proteolytic cleavage, with its activity being precisely regulated by tissue inhibitors of metalloproteinases (TIMPs). Dysregulation of MMP-2 has been linked to a variety of pathological conditions, including cardiovascular diseases, diabetic complications, kidney diseases, and cancer. In cardiovascular diseases, it contributes to vascular remodeling, atherosclerosis, and aneurysms, while in fibrotic diseases, it mediates excessive ECM degradation leading to tissue scarring. In diabetes, elevated MMP-2 activity exacerbates complications such as nephropathy, retinopathy, and cardiovascular disease. In cancer, MMP-2 facilitates tumor invasion and metastasis by degrading ECM components and promoting angiogenesis. Despite its essential roles in both physiological and pathological processes, targeting MMP-2 for therapeutic purposes presents challenges due to its dual functions in tissue remodeling and repair, raising concerns about unplanned consequences such as impaired tissue healing or excessive tissue damage. These challenges underscore the need for future research to focus on developing selective modulators that can precisely balance their activity under specific disease environments. Clinical trials targeting MMP-2 modulation highlight the potential of gelatinase inhibitors, including those targeting MMP-2, to reduce tumor progression in fibrosarcoma, breast, and lung cancers. This paper reviews the structure, function, and regulation of MMP-2, its involvement in disease pathogenesis, and the potential challenges in the therapeutic implications of modulating its activity.

## 1. Matrix Metalloproteinase-2 (MMP-2): Structure, Functions, and Roles in Disease

Matrix metalloproteinases (MMPs), also known as matrixins, are a family of calcium-dependent zinc-containing enzymes essential for the degradation and remodeling of the extracellular matrix (ECM—structural network supporting tissues and organs) [1,2]. This family consists of 19 different enzymes, which are commonly classified based on their substrates and the organization of their structural domains. The main subfamilies are collagenases, gelatinases, stromelysins, membrane-type MMPs, and other MMPs [3]. These endopeptidases are master regulators of tissue remodeling, regulating biological processes such as wound healing, inflammation, and tissue regeneration. MMPs could also influence endothelial cell function as well as smooth muscle cell migration, cell proliferation, calcium signaling, and contraction [4]. Dysregulation of MMPs contributes to pathological conditions like cardiovascular diseases, cancer, chronic renal and pulmonary diseases, and inflammatory disorders, where they augment tissue destruction, tumor invasion, and altered tissue regeneration and repair mechanisms [1,2,3,4,5].

Among the MMP family, MMP-2 (also known as gelatinase A) is noticeable due to its prominent role in ECM remodeling. MMP-2, along with MMP-9 (gelatinase B), belongs to the gelatinase subgroup and is characterized by its ability to degrade type IV and V collagen, fibronectin, laminin, elastin, along with other ECM proteins [6]. Under normal physiological conditions, MMP-2 contributes to the breakdown of the extracellular matrix, playing essential roles during processes like embryonic development and tissue remodeling. MMP-2 is a 72 kDa enzyme composed of several distinct structural domains. The chemical structure of MMP-2 consists of a zinc ion at the catalytic site, essential for its proteolytic activity, and a conserved catalytic domain (active site facilitating enzymatic reactions) responsible for substrate recognition and cleavage. MMP-2 exists in two isoforms: the full-length isoform, which includes a pro-domain, catalytic domain, and hemopexin-like domain, and the N-terminal truncated isoform, which lacks part of the pro-domain and is constitutively active. MMP-2 exists in various isoforms, including the inactive proMMP-2, the active enzyme capable of ECM degradation, and membrane-bound forms that influence cell migration and invasion. Alternative splicing also generates different isoforms with unique structural characteristics, which can modulate their activity and tissue-specific roles in both physiological processes and disease progression. MMP-2 exhibits physical properties typical of metalloproteinases, including a globular structure and the ability to cleave a broad spectrum of substrates, such as extracellular matrix components and cytokines [7,8]. MMP-2 is secreted as an inactive pro-enzyme (proMMP-2) that requires activation before it can exert its proteolytic activity. The activation typically occurs through the proteolytic cleavage of the pro-peptide region by other MMPs, such as MMP-14 (MT1-MMP) or plasmin. These enzymes cleave the pro-domain, releasing the active form of MMP-2, which can then degrade its target ECM substrates [2,3,4]. The activity of MMP-2 is tightly controlled by tissue inhibitors of metalloproteinases (TIMPs), primarily TIMP-2. TIMPs bind to the active form of MMP-2 in a 1:1 stoichiometric manner, blocking the catalytic site and preventing ECM degradation. This regulatory balance between MMPs and TIMPs is crucial in maintaining ECM homeostasis and preventing pathological tissue remodeling. Genomic data analysis has revealed associations between MMP-2 gene polymorphisms and various diseases, including dilated cardiomyopathy, highlighting the genetic influence on disease susceptibility and progression. Furthermore, studies have shown that MMP-2 expression correlates with other genes like lipoxygenase (*Lox*) and *Col1a1*, indicating its involvement in complex molecular networks underlying diseases such as breast cancer or periodontitis [9,10].

Recently, MMP-2 has been shown to interact with many functional proteins, uncovering its prominent role in health and disease. As evidence, we can enumerate several distinct roles of MMP-2, including results published by Terni and Ferrer who show that MMP-2 can cleave recombinant tau protein in vitro in a dose-dependent manner, indicating its involvement in normal protein *tau* proteolysis [11]. Research has shown that MMP-2 deficiency leads to inhibition of the activation of transforming growth factor-β (TGF-β) and the Smad2/3 pathway, emphasizing the interaction between MMP-2 and other signaling proteins involved in the progression and occurrence of thoracic aortic aneurysm [12]. MMP-2 also facilitates epidermal growth factor receptor (EGFR) signaling by promoting the release of EGFR ligands as well as regulates the canonical inflammasome pathway leading to the release of interleukin-1β and interleukin-8 that controls inflammatory processes at different levels [13]. Also, the possibility of the cleavage of TNF-α, a potent pro-inflammatory and immunomodulatory cytokine implicated in inflammatory conditions, by MMP-2 suggests a pivotal role for MMP-2 in homeostasis [14]. Additionally, in cancer, MMP-2 is involved in promoting invasion and metastasis by facilitating the vascular endothelial growth factor (VEGF), insulin-like growth factor (IGF), and TGF-β dependent pathways [15].

MMP-2 has gained renewed attention in recent years due to the development of selective MMP-2 inhibitors, including small molecules and monoclonal antibodies, which might overcome the challenges of off-target effects and toxicity seen in the past with traditional wide-spectrum inhibitors [16]. Furthermore, innovative technologies such as nanomedicine, CRISPR-based gene editing, and advanced drug delivery systems have facilitated new approaches for precisely modulating MMP-2 activity, enabling localized treatment within the targeted tissues and minimizing side effects by preserving healthy processes [17,18].

Further exploration of MMP-2 interactions with other proteins may reveal novel therapeutic targets for modulating MMP-2 activity in diverse pathological conditions, as described in the next paragraphs.

## 2. The Roles of MMP-2 in Cardiovascular Diseases (CVDs)

MMP-2 is involved in multiple cardiovascular conditions such as aortic aneurysm formation, atherosclerosis, chronic thromboembolic pulmonary hypertension (CTEPH), and myocardial fibrosis [19]. An aneurysm is characterized by the degradation and functional loss of elastin in the aortic media. MMP-2 plays a crucial role in weakening ECM components such as elastin, collagen, fibronectin, and proteoglycans, thereby compromising the integrity of the aortic wall [20]. Moreover, TIMP-2 knockout attenuates aneurysm progression due to playing a role in MMP-2 activation. Transgenic animal models for MMP-2 exhibit enlarged mid-ventricular coronary luminal areas, along with areas of aneurysmal dilation, ectasia, and perivascular fibrosis [21]. In atherosclerosis, MMP-2 degrades ECM components within the arterial walls, contributing to plaque destabilization and rupture. This degradation weakens the structural integrity of the arterial wall, making it prone to aneurysm formation, which can lead to vessel rupture and life-threatening events like stroke or heart attack. Furthermore, MMP-2 is involved in the extravasation of inflammatory cells (i.e., macrophages and T-cells) into the arterial wall. These cells release pro-inflammatory cytokines and additional matrix-degrading enzymes, further promoting ECM degradation and enhancing atherosclerotic plaque growth [22]. At the same time, ECM degradation and the inflammatory environment can lead to reduced production of nitric oxide (NO), a key vasodilator and endothelial protector. This process further impairs endothelial function and promotes a pro-atherogenic state [23,24]. Recent studies have shown that reduced NO bioavailability, together with increased oxidative stress, enhances MMP-2 activity, contributing to vascular dysfunction and disease progression. In conditions like preeclampsia, elevated oxidative stress and inflammation further upregulate *Mmp2* through cytokines like interleukin-8, linking NO deficiency to increased *Mmp2* expression and vascular remodeling [25,26]. The last factor leading to promoting atherosclerosis by MMP-2 is augmented pro-inflammatory signaling due to the release of ECM degradation products like TNF-α, interleukins, TGF-β, monocyte chemoattractant protein-1 (MCP-1) as well as MMP-9, which further degrades ECM components and amplifies inflammation, contributing to endothelial layer dysfunction [27]. The role of MMP-2 in CTEPH has been widely studied, giving evidence of the negative role of MMP-2 through its involvement in ECM remodeling, endothelial dysfunction, smooth muscle cell proliferation, and vascular remodeling [28]. Similarly to previously discussed mechanisms, MMP-2-induced ECM degradation leads to endothelial cell detachment, loss of endothelial integrity, and an increase in vascular permeability. This pathology disrupts the physiological endothelial barrier integrity, promoting inflammatory cell infiltration, particularly macrophages and lymphocytes. The inflammatory *milieu* results in the release of pro-inflammatory cytokines and growth factors, including VEGF and TGF-β, which further accelerate the fibrotic process and smooth muscle proliferation in the pulmonary vasculature. Another role of MMP-2 in CTEPH is its contribution to the proliferation of vascular smooth muscle cells (SMCs) within the intima and media of pulmonary arteries, which leads to further impairment of artery function within pulmonary microcirculation [29,30]. The role of MMP-2 in the progression of myocardial fibrosis has been linked to, similarly to other CVDs, the excessive degradation of ECM. Under physiological conditions, the activity of MMP-2 is tightly regulated by various factors, namely TIMPs (with the prominent role of TIMP-2), reversion-inducing cysteine-rich protein with kazal motifs (RECK, which acts as an MMP-2 suppressor), or membrane-type 1 matrix metalloproteinase (MT1-MMP), which is essential for activating pro-MMP-2 on the cell surface [31,32]. When these factors are not capable of sufficient inhibition of MMP-2 activity, pro-inflammatory factors and dysregulation in redox balance come into play, leading to progressive fibrosis within the heart muscle tissue. Over time, it results in a fully developed clinical picture of myocardial fibrosis [33]. This paragraph summarized the multifaced role of MMP-2 in CVD progression and occurrence. It is worth noting that under CVD-like conditions, MMP-2 is often dysregulated, leading to either excessive breakdown or inadequate turnover of ECM components. This imbalance very likely results in pathological changes as described: myocardial fibrosis, ventricular dilation, and vascular remodeling, all of which compromise cardiovascular function and can accelerate disease progression. Thus, MMP-2 activity of ECM degradation directly influences the development, severity, and progression of a plethora of cardiovascular pathologies, including heart failure, hypertension, and atherosclerosis, which are further related to an inflammatory state, redox disbalance, and altered adaptive immunity functions [34,35].

## 3. MMP-2 Impacts the Course of Diabetic Complications

Type 1 diabetes mellitus (T1DM), also referred to as juvenile-onset diabetes or insulin-dependent diabetes mellitus, is a chronic autoimmune disorder marked by targeted destruction of insulin-producing beta cells in the pancreatic islets of Langerhans. This autoimmune-mediated beta-cell destruction results in absolute insulin deficiency, leading to hyperglycemia. While T1DM commonly manifests in childhood or adolescence, it can present at any age [36,37]. Ongoing research on T1DM focuses on unraveling its complex pathogenesis, identifying genetic and environmental risk factors, and developing innovative therapies. Current investigational approaches include immunotherapies designed to modulate or halt the autoimmune response responsible for beta-cell destruction. These therapies aim to preserve residual beta-cell function, delay disease onset, and potentially prevent the progression of T1DM. One of the potential targets investigated within the last decade is MMP-2 [38].

During T1DM, MMP-2 appears to contribute to the pathogenesis of diabetic complications through its involvement in specific signaling pathways. Studies indicate that MMP-2 can be activated by factors such as insulin-like growth factor-2 (IGF-2) and VEGF. These factors initiate MMP-2 activation via the PI3-K, protein p38, and JNK signaling pathways [39]. Additionally, intracellular MMP-2 has been shown to play a role in regulating platelet activation by PAR1-dependent Gq and G12/13 pathway activation leading to platelets hyperactivity that might further worsen diabetic vasculopathy [40]. Through these mechanisms, MMP-2 may influence vascular and platelet-related complications commonly associated with T1DM. Furthermore, MMP-2 has been linked to excessive cell migration and invasion through the regulation of epithelial–mesenchymal transition (EMT) and EGFR-mediated signaling pathways that are also tightly connected to diabetic liver disease as shown in the hepatic mesenchymal culture model [41]. The axis MMP-2-EMT/EGFR seems to be dependent on miR-26a-5p, which negatively regulates cadherin and promotes cadherin 1 expression in diabetic liver disease. In another study, enhanced cardiac expression of two isoforms of *Mmp2* was observed in an experimental diabetic heart model. The researchers hypothesized that high glucose stimulation induced the expression of full-length MMP-2 and N-terminal truncated MMP-2 in vitro and in diabetic heart models, suggesting a potential link between *Mmp2* isoforms and diabetic cardiomyopathy [42]. Another interesting insight into vascular complications from diabetes was provided by Liu et al. [43]. They showed that the forkhead box protein O1 (FoxO1—a key regulator of cellular metabolism and an early predictor of CVDs) is significantly upregulated in carotid arteries in the T1DM rat model, accompanied with adverse vascular remodeling described as increased wall thickness, a carotid medial cross-sectional area, a media-to-lumen ratio, and a decreased carotid artery lumen area. Simultaneously, increased levels of FoxO1 were associated with elevated levels of MMP-2, suggesting the existence of an interrelation between these two proteins in promoting T1DM-induced vasculopathy. Another matter of progressing T1DM is gradually decreasing renal function. MMP-2 was shown to be a predictor of diabetic renal fibrosis leading to the progression of chronic kidney disease. This pathology was associated with TGF-β1 signaling and correlated with ERK1/2 expression and modulation of MMPs/TIMPs expression [44]. Shiau et al. showed that *Mmp2* expression and activities are significantly increased in patients with T1DM, and they suggested that these levels are elevated even before the onset of complications in diabetic patients [45]. Furthermore, as evidence of the role of MMP-2 in T1DM progression, MMP-2 was classified as a novel marker of neurovascular complications in T1DM [46]. On the other hand, pediatric studies have shown that urine levels of MMP-2 and its ratio to creatinine could not be used as predictors of fibrosis in the early development of T1DM in children [47]. On the contrary, MMP-2 seems to be a very useful marker of microangiopathies under T1DM conditions in adults and children [48].

Another type of DM—type 2 diabetes mellitus (T2DM)—accounts for approximately 90% of all diabetes cases. It is hallmark is insulin resistance, where the hormonal response to circulating insulin is reduced. Initially, this resistance is counteracted by increased insulin production by the pancreas to maintain glucose balance, but progressively, insulin production declines, leading to T2DM. T2DM is most prevalent in individuals over 45 years old and it has been correlated with growing rates of excessive body weight, sedentary lifestyles, and consumption of high-energy diets [49,50]. There is evidence that MMP-2 plays an important role in the initiation, progression, and recurrence of T2DM-linked complications. Firstly, higher MMP-2 levels were linked to persistently higher levels of high-sensitive C-reactive protein (hs-CRP), suggesting the role of MMP-2 in chronic inflammation, which is a common feature of T2DM [51]. Similar observations of the involvement of MMP-2 in interleukins-6/20-dependent pro-inflammatory responses were made by Lv in elderly patients suffering from T2DM [52]. Furthermore, thioredoxin-interacting protein, playing a role in pancreatic β-cell dysfunction and upregulating the inflammatory response in hyperglycemia, was found strongly associated with several predictors of severe T2DM (ICAM-1, MMP-2, and P-selectin) [53]. Similarly, to deteriorate the role of MMP-2 in CVD progression in T1DM, MMP-2 facilitates the progress of CVD in T2DM. Preil et al. suggested that the diabetic environment affects the circulating amounts of MMP-2 and might promote peripheral arterial disease [54]. On the other hand, in the same study, no connections between the levels of MMP-2 and myocardial ischemia, increased carotid thickness, and decreased ankle–brachial blood pressure were found [54]. The possible explanation of the divergent MMP-2 role in CVD is its polymorphism in the T allele of *Mmp2* C(-1306)T. It has been shown that T2DM patients carrying this allele have a significantly reduced risk of CVD when the same allele is associated with susceptibility to stroke in these patients [55]. Another genetic difference related to the risk of vasculopathy occurrence during T2DM was proven by Sarray and colleagues. In their work, they showed that *Mmp2* variants rs243864 and 243866 are related to the susceptibility to diabetic retinopathy and the progression of the disease in the population with T2DM [56]. In T2DM, MMP-2 is impacted by factors such as dyslipidemia, oxidative stress, and inflammation, which contribute to endothelial injury and plaque destabilization. Vasculopathies in T1DM primarily result from prolonged hyperglycemia leading to microvascular damage, affecting organs such as the kidneys and eyes, while in T2DM, vasculopathies are more closely associated with macrovascular complications, including atherosclerosis and peripheral artery disease, exacerbated by insulin resistance, dyslipidemia, and systemic inflammation [57]. Understanding the cellular pathways involving MMP-2 in the context of diabetes can provide insights into the mechanisms underlying diabetic complications and potential therapeutic targets for intervention. Further research is needed to elucidate the specific roles of MMP-2 in diabetes-related pathologies and to explore its potential as a target for therapeutic strategies.

## 4. The Involvement of MMP-2 in the Progression of Renal Function Impairment

As MMP-2 has been identified as a key player in fibrotic diseases and ECM degradation, its role in both acute and chronic kidney disease has been widely studied. Acute kidney injury (AKI) is a clinical syndrome characterized by a rapid decline in glomerular filtration rate leading to accumulation of metabolic waste products [58]. Oxidative stress and inflammatory milieu are common features in models of AKI induced by ischemia–reperfusion (I-R) injury [59]. Ceron et al. showed that NH2-terminal truncated MMP-2 leads to tubular cell necrosis, inflammation, and fibrosis within the renal system. They suggested that this form of MMP-2 triggers the kidney to enhance susceptibility to I-R injury via induction of mitochondrial dysfunction, leading to AKI [59]. The involvement of MMP-2 in AKI was further supported in a study by McNair, who demonstrated that the serum and urine levels of activity of MMP-2 are associated with the clinical endpoint of AKI and seem to have earlier rising levels as compared with those of serum creatinine [60]. Interestingly, MMP-2 is not only a possible marker of AKI severity as well as a driver of this pathology. It is also involved in recovery after tubular damage during AKI. Mice with *Mmp2* knockout were characterized by impaired proliferation of tubular epithelial and damaged tubules that were covered with elongated and immature regenerated epithelial cells after AKI. Furthermore, this incomplete recovery of injured microvasculature was also related to persistent macrophage infiltration [61]. Also, TIMP-2 was shown to be a novel prognostic factor to determine the severity and prognosis of AKI under different clinical settings [62,63].

Recent studies have shed light on the role of MMP-2 in acute kidney injury (AKI) and its transition to chronic kidney disease (CKD), emphasizing its involvement in maladaptive repair mechanisms. Sharma et al. showed recently that TGF-β1/SMAD3 pathway activation and increased collagen expression (which are tightly interrelated to MMP-2 activity) exacerbated AKI and its transition into CKD [64]. Furthermore, Wyczanska et al. provided evidence that MMP-2 plays a role in urinary tract obstruction during renal development leading to inflammation, tubular apoptosis, and interstitial fibrosis. These conditions might also promote the development of CKD [65].

CKD is a progressive disease with high morbidity and mortality that occurs commonly in the general adult population, especially in people with diabetes and hypertension [66,67]. MMP-2 is involved in ECM turnover in the glomeruli and tubulointerstitium, and its elevated levels are associated with CKD progression, particularly among those with low inflammation and those with proteinuria [68]. MMP-2 was also shown to promote the development of CKD through the various interactions with tumor necrosis factors, monocyte chemoattractant proteins, and reactive oxygen species disbalance [69]. Interestingly, higher levels of MMP-2 and TIMP-2 were found in serum from patients with CKD, and serum levels of MMP-2 were correlated with the degree of kidney failure [70]. MMP-2 due to activation of pro-inflammatory cytokines and chemokines can also amplify the renal tissue damage during the late stages of CKD. Recent studies have shown that MMP-2 interacts with TGF-β signaling pathways, a pivotal trigger of fibrotic processes, promoting ECM deposition and the activation of fibroblasts. This interplay accelerates glomerulosclerosis and tubulointerstitial fibrosis, which are hallmark features of CKD, thereby impairing renal function and contributing to disease development. MMP-2 dysregulation in CKD has been linked to increased levels of fibrotic markers such as collagen and α-SMA, highlighting its role as a potential therapeutic target for fibrosis in renal diseases [71,72,73]. Based on the multifaced functions, MMP-2 was also proposed as a balancing factor during the renal impairment progression. A study by Takamiya et al. showed that renal expression and activity of MMP-2 are increased as a compensatory mechanism in the early phase of diabetic nephropathy. Furthermore, they suggested that MMP-2 could be considered as having a protective role in the progression of CKD [74]. The role of MMP-2 was also suggested in renal carcinoma; however, the exact role seems to depend on the stage of the carcinogenic process, showing how multifaced the role MMP-2 has [75]. MMP-2 mediates the degradation of the glomerular basement membrane (GBM), leading to compromised structural integrity. This disruption augments the leakage of proteins into the urine, resulting in proteinuria. Furthermore, the breakdown of GBM components contributes to podocyte injury, impairing their function in maintaining the filtration barrier. This cascade of events exacerbates glomerulosclerosis, ultimately advancing renal pathology and contributing to the progression of kidney diseases such as diabetic nephropathy [66]. MMP-2 activity exhibits distinct patterns across the stages of kidney function impairment, with varying roles in the progression of renal disease. In the early stages of CKD, MMP-2 is involved in tissue repair and ECM remodeling, facilitating the healing of renal injury by degrading damaged ECM components. However, its activity is well regulated to prevent excessive ECM degradation that could lead to more severe tissue injury. During CKD progress, dysregulation of MMP-2 takes place, leading to its overexpression and excessive ECM degradation, which contributes to the development of glomerulosclerosis and tubulointerstitial fibrosis. This imbalance in MMP-2 activity accelerates renal fibrosis, impairing kidney function and facilitating the progression of CKD to end-stage renal disease [67]. Currently, the non-proteolytic function of MMP-2 is a rapidly evolving topic of research, which is also associated with the progression of CKD. Therefore, more bench-to-bedside studies are needed to fully establish the role of MMP-2 under impaired renal function.

## 5. The Multiple Roles of MMP-2 Across Different Biological Systems

**Figure 1:**
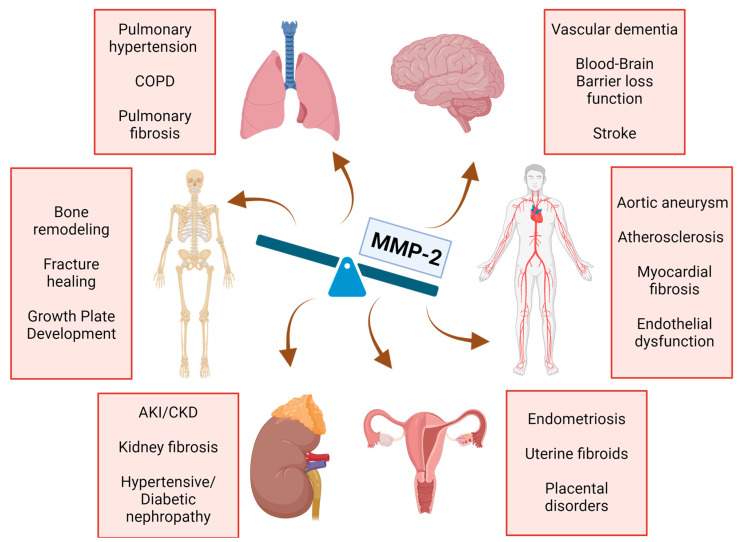
The multifaceted effects of MMP-2 activity imbalance on multiple organs illustrate its key role in both physiological and pathological processes (Shown by arrows). Dysregulated MMP-2 activity can lead to tissue remodeling, fibrosis, and damage across various organs, including the kidneys (via glomerulosclerosis and proteinuria), the cardiovascular system (contributing to vascular remodeling and atherosclerosis), and bone metabolism (due to osteoclast activity modulation). MMP-2—metalloproteinase-2; COPD—chronic obstructive pulmonary disease; AKI—acute kidney injury; CKD—chronic kidney disease.

The true multifaced nature of MMP-2 (Figure 1) could be revealed when discussing the recent advances in basic science research. MMP-2 plays an important role in maintaining the reproductive system homeostasis. MMP-2 is integral to cyclic endometrial changes during the menstrual cycle and implantation. Moreover, the levels of MMP-2 are highly elevated in the ectopic endometrium of women with visible endometriotic lesions and eutopic endometrium in patients with no signs of endometriosis [76]. A study by Deady et al. showed the role of follicular adrenergic signaling in *Mmp2* activation and ovulation in *Drosophila*, which is likely conserved in other species [77]. Moreover, MMP-2 activity in seminal plasma has a positive effect on sperm count and motility. The role of MMP-2 in follicular fluid and seminal plasma could be an important factor in embryo quality in patients undergoing successful intracytoplasmic sperm injection (ICSI) and may affect the outcome of ICSI [78]. The intraovarian role of MMP-2 includes ECM remodeling during folliculogenesis, follicle atresia, and postovulatory regression [79]. Recently, Kalev-Altman showed a pivotal role for MMP-2 in myometrium remodeling during the mammalian parturition process, underlining a novel cause for dystocia due to a loss in MMP-2 activity in the uterine tissue [80].

In the nervous system, the role of MMP-2 goes far beyond its detrimental roles and is a master regulator in many developmental events in the nervous system as well as promoting regeneration and repair of the injured nervous system [81]. MMP-2 was shown to control multiple synaptic plasticity-related processes, namely dendritic spine development, cell adhesion, neurite guidance, and cell migration during the development of the central nervous system (CNS) [82,83]. Similarly, studies in *Drosophila* using RNAi-mediated knockdowns and overexpression of TIMP have revealed that *Mmp2* exerts spatial regulation over FGF signaling, which governs the branching morphogenesis of the developing air sac [84]. Moreover, MMP-2 has a potential role in the activation of neuroinflammatory pathways and neurosignaling components as well as might promote compromising vascular integrity resulting in barrier leakage (e.g., cerebrovascular membrane barrier) [85]. Song et al. showed that MMP-2 activity, specifically at the border of the CNS parenchyma, strongly enhances the leukocyte transmigration process. It reveals the previously unknown role of MMP-2 in controlling cells and cytokine migration through the blood–brain barrier (BBB) [86]. These findings were recently expanded by showing that ischemia-induced secretion of MMP-2 may contribute to early BBB disruption in ischemic stroke via interrupting the shared Scube2-Shh pathway between brain capillary endothelial cells and perivascular astrocytes [87].

**Figure 2:**
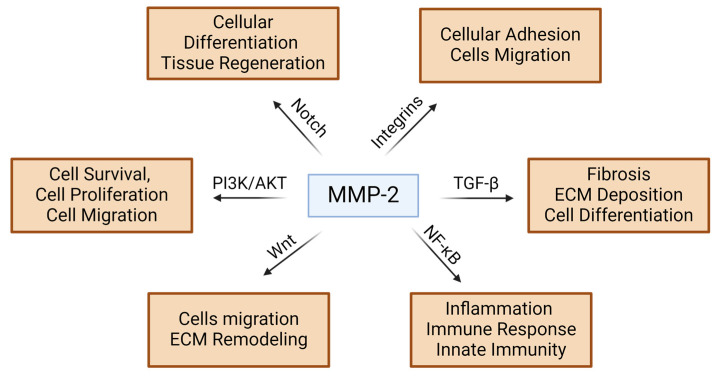
The multifaceted nature of MMP-2 biology is driven by different pathways leading to various biological and pathological phenomena. MMP-2—metalloproteinase-2; ECM—extracellular matrix; TGF-β—Transforming Growth Factor Beta; NF-κB—Nuclear Factor kappa-light-chain-enhancer B; PI3K/AKT—Phosphatidylinositol 3-kinase/AKT pathway.

The role of MMP-2 in the cancer environment has been proposed decades ago. However, recent years brought many new aspects to the understating of these phenomena. MMP-2 facilitates tumor invasion and metastasis by degrading basement membrane components, enabling cancer cell migration and tube formation, critical for neovascularization in tumors [88]. It is also possible that aberrant MMP-2 activity contributes to resistance mechanisms against anti-angiogenic therapies [89]. MMP-2 has been found to play a role in tumor invasion and metastasis, particularly in glioma progression, where it interacts directly with the fibronectin receptor α5β1 integrin [90,91]. MMP-2 has been also implicated in the progression of gastric cancer by promoting metastasis through the phosphorylation of activating transcription factor 1 (ATF1) [92]; however, the action of MMP-2 is not associated with the mTOR pathway [93]. Clinical data have shown that *Mmp2* is upregulated and positively correlated with the expression of the long lncRNA P73 antisense RNA 1T (TP73-AS1) in ovarian cancer tissues, and knockdown of *Mmp2* attenuates the effects of TP73-AS1 overexpression on cell invasion and migration [94]. MMP-2 has been shown to be activated in a cancer-associated fibroblast-conditioned medium, leading to increased invasion of keratinocytes in a TGF-β-dependent manner. Furthermore, MMP-2 plays an emerging role in the regulation of cancer-associated fibroblast infiltration, potentially participating in immunotherapy response [95]. Invasion and metastatic potential of melanoma cells is driven, i.e., by MMP-2 on hypoxia-dependent pattern. Recent studies showed a new, CD147-related mechanism to induce MMP-2 in multiple cancers. Importantly, knocking down CD147 attenuates MMP-2 response to hypoxia in melanoma cell lines, confirming the new mechanism [96]. Interestingly, MMP-2 activation by membrane type-1-MMP potentially amplifies protease activity and combination with direct cleavage of substrate causes effective tissue degradation and enhances tumor invasion and metastasis [97] (Table 1, Figure 2).

MMP-2 also plays a role in maintaining homeostasis of the musculoskeletal system, and its overexpression might lead to pathological outcomes. In the bone, many functions were assigned to MMPs, including osteoblast/osteocyte differentiation, solubilization of the osteoid in bone resorption, osteoclast recruitment, and migration and as a coupling factor in bone remodeling under physiological conditions [98]. MMP-2 is important for the differentiation and survival of osteoblasts as well as required for proper osteocyte lacunae formation and osteocytic perilacunar/pericanalicular remodeling (collagenase activity) [98]. Jiang et al. showed for the first time that MMP-2 inhibitor 1 (MMP-2-I1) has a positive role in the osteogenesis of human bone marrow mesenchymal stem cells (hBMSCs) and angiogenesis of human vascular endothelial cells (HUVECs). This action seems to be dependent on MMP-2-I1 activation of the p38/mitogen-activated protein kinase signaling pathway [99]. Furthermore, MMP-2 seems to alter bone growth by impacting osteoclast and osteoblast activity and proliferation [100]. Knockout of the *Mmp2* gene in mice model had a direct effect on osteopontin (which promotes osteoclast activity) and sialoprotein expression, which has been proven to promote osteoblast development and activity. As a result of the *Mmp2* knockout, enhanced bone reabsorption or bone growth was observed in these mice [101,102]. Interestingly, mutations in genes encoding MMP-2 incur severe bone abnormalities in humans. Based on this evidence, we can hypothesize that *Mmp2* mutations that cause loss of function have an impact on osteoblasts’ energy metabolism, limiting their ability to create bone during regeneration processes [103]. The above-mentioned data suggest also that MMP-2 plays a major protective role in osteogenesis and bone regeneration processes.

Genetic polymorphism in the *Mmp2* gene significantly influences its function and is implicated in the divergent pathogeneses of diseases [104,105]. One of the most studied variants is the -1306 C/T polymorphism in the promoter region, which modulates the transcriptional activity of *Mmp2* and alters its expression levels. Individuals carrying the T allele have been shown to exhibit higher MMP-2 activity, which can exacerbate conditions characterized by excessive ECM degradation, such as cancer metastasis, osteoarthritis, and CVD [50]. Furthermore, the -1306 T allele has been associated with an increased risk of myocardial infarction and stroke, as elevated MMP-2 activity can lead to vascular remodeling and plaque destabilization. On the contrary, polymorphisms such as rs243865, which reduce *Mmp2* expression, are associated with impaired ECM remodeling and tissue repair, contributing to diseases such as diabetic nephropathy, pulmonary fibrosis, and chronic wounds [106]. In osteoarthritis, the rs243849 polymorphism has been linked to increased MMP-2 levels, accelerating cartilage degradation. These genetic variants affect disease susceptibility and also modulate disease severity and progression. For example, in cancer, the overexpression of *Mmp2* due to specific polymorphisms promotes tumor invasion and metastasis, particularly in breast and lung cancer [107]. Furthermore, the presence of *Mmp2* polymorphisms may influence the efficacy of therapeutic strategies targeting the enzyme, such as MMP inhibitors or even gene therapies. However, the clinical implications of these polymorphisms in therapeutic outcomes remain unexplored. To better understand their functional consequences, future research should focus on detailed molecular characterization of these variants using in vivo knockout/knockdown models to assess their impact on disease pathophysiology. Moreover, advanced gene-editing technologies such as CRISPR may offer potential advances for therapeutic intervention and enable modulation of MMP-2 activity in patients with disease-associated polymorphisms.

**Table 1:** The summary of the signaling pathways involved in MMP-2-mediated pathological processes. The diseases listed are linked to specific signaling pathways that regulate key mechanisms such as fibrosis, inflammation, cell apoptosis, and tissue remodeling. The associated references provide further information on the signaling pathways and their roles in disease progression. TGF-β: Transforming Growth Factor Beta; PI3K/AKT: Phosphoinositide 3-Kinase/Protein Kinase B; MAPK/ERK: mitogen-activated protein kinase/extracellular signal-regulated kinase; VEGF: vascular endothelial growth factor; RAGE: Receptor for Advanced Glycation End Products; NF-κB: Nuclear Factor Kappa B; SMAD: Small Mothers Against Decapentaplegic; ET-1: Endothelin-1; AGES: Advanced Glycation End Products; PKC: Protein Kinase C; RAAS: Renin–Angiotensin–Aldosterone System; HIF: Hypoxia-Inducible Factor; EGF/EGFR: epidermal growth factor/epidermal growth factor receptor; RANK/RANKL/OPG: Receptor Activator of NF-κB/RANK Ligand/Osteoprotegerin; JAK/STAT: Janus Kinase/Signal Transducer and Activator of Transcription.

Disease/Condition	Signaling Pathways Affected	Effect of MMP-2	References
Cardiovascular Diseases	TGF-β, PI3K/AKT, MAPK/ERK, Integrin, VEGF	Aneurysm, Vascular Remodeling and Calcification, Reperfusion Injury, Atherosclerosis, Arterial Stiffness, Hypertension, Heart Valve Disease	[16,17,18,19,20,21,22,23,24,25,26,27]
Diabetic Cardiomyopathy	PI3K/AKT, RAGE, NF-κB, MAPK/ERK, TGF-β/SMAD, ET-1, Inflammasome Pathway	Cardiac Fibrosis and Hypertrophy, Oxidative Stress, Myocardial Contractile Dysfunction, Cardiomyocyte Apoptosis, Propagation of Inflammation	[35,36,37,108,109]
Diabetic Nephropathy	TGF-β/SMAD, PI3K/AKT, AGEs/RAGE, NF-κB, PKC	Glomerular Basement Membrane (GBM) Thickening, Tubulointerstitial Fibrosis, Injury and Loss of Podocytes, Propagation of Inflammation and Oxidative Stress	[46,47,110]
Diabetic Retinopathy	VEGF, NF-κB, TGF-β, Integrin	Loss Of Blood–Retinal Barrier, Retinal Capillary Basement Membrane Thickening	[111,112]
Acute Kidney Injury	TGF-β, PI3K/AKT, MAPK/ERK, NF-κB, RAAS, HIF	Tubular Cell Apoptosis, Impaired Tubular Cells Repair, Propagation of Cytokine Release, Acute Inflammation	[53,54,55,56,57,58,113]
Chronic Kidney Disease	TGF-β, PI3K/AKT, MAPK/ERK, NF-κB, RAAS, Notch	Renal Fibrosis, Podocyte Injury and Glomerular Permeability, Tubulointerstitial Fibrosis, Vasculopathy, Persistent Inflammation, and Imbalanced Oxidation	[60,61,62,63,64,113]
Neurodegenerative Diseases	TGF-β/SMAD, NF-κB, PI3K/AKT, Notch, AGEs/RAGE	Blood–brain Barrier Disruption, Neuroinflammation, Glial Activation, Direct Neuronal Injury, Propagation of Inflammation	[69,70,71,72,73,74,75]
Cancer	TGF-β, PI3K/AKT, MAPK/ERK, VEGF, HIF, Wnt/β-catenin, NF-κB, Integrin, EGF/EGFR	Tumor Invasion and Metastasis, Remodeling the Tumor Microenvironment, Pathological Growth Factors Stimulation, Angiogenesis, Increased Tumor Cell Survival	[77,78,79,80,81,82,83,84,85,86]
Bone Remodeling Disorders	TGF-β, PI3K/AKT, MAPK/ERK, Wnt/β-catenin, JAK/STAT, RANK/RANKL/OPG	Osteoclast-Mediated Bone Resorption, Osteoporosis, Incomplete Bone Regeneration, Osteoarthritis and Cartilage Degradation	[87,88,89,90,91,92,114]

## 6. Limitations of the Treatments Harboring on MMP-2

Several studies showed that targeting MMP-2 holds therapeutic promise, albeit there are several challenges in developing specific inhibitors due to the enzyme’s role in both physiological and pathological processes. MMPs, in general, are critical for ECM remodeling, wound healing, and angiogenesis, so their inhibiting could potentially interfere with these essential functions. The limitations of MMP-2 inhibitors, including off-target effects and toxicity, have spurred interest in alternative strategies such as gene editing and isoform-selective targeting. Gene editing techniques (e.g., CRISPR) enable precise modifications at the genetic level, allowing for the selective modulation of *Mmp2*/MMP-2 expression/activity. Additionally, isoform-selective inhibitors offer the potential to target specific MMP-2 isoforms, minimizing side effects and enhancing therapeutic efficacy. Gene editing tools, such as CRISPR, enable precise targeting of *Mmp2* expression at the genetic level, offering a solution to its ubiquitous presence and minimizing off-target effects seen with wide-spectrum inhibitors. Additionally, isoform-selective inhibitors can modulate specific MMP-2 isoforms, reducing systemic toxicity and enhancing therapeutic efficacy in targeted tissues or organs. A targeted approach is necessary to selectively modulate MMP-2 activity without disrupting its beneficial roles in the human body. Another challenge is the existence of different isoforms of MMP-2. The full-length and truncated isoforms of MMP-2 exhibit distinct functional properties, contributing to tissue-specific remodeling and fibrosis. Considering this, designing inhibitors that selectively target specific isoforms of MMP-2 is crucial to avoid unintended effects on normal tissue function. Furthermore, the regulation of MMP-2 is influenced by numerous signaling pathways, including the PI3K/AKT pathway, p38 MAPK, and JNK signaling. These pathways are activated by growth factors such as VEGF, TGF-β, and IGF, all of which are elevated in various pathological conditions. The interaction of MMP-2 with these signaling pathways complicates the development of inhibitors, as it requires a detailed understanding of the specific mechanisms that modulate *Mmp2* expression and activity in different tissues [115,116]. The complex biological mechanisms underlying MMP-2 activity and regulation highlight the challenges associated with targeting this enzyme for therapeutic purposes. Despite these challenges, there are clinical trials focusing on modulating MMP-2 activity. Study NCT05670834 investigates the potential association between *Mmp2* gene polymorphism and susceptibility to cataract development [117]. Another study (NCT04773028) focused on the role of MMP-2 in pulmonary arterial wall remodeling in patients with chronic thromboembolic pulmonary hypertension [118]. Another ongoing study examines the interconnection between relaxin, MMP-2, and IL-6 in women with chronic pelvic pain [119]. MMP-2 inhibition remains a promising strategy for treating diseases characterized by excessive ECM remodeling, particularly in diabetic nephropathy and cardiovascular disease. Further research into the molecular mechanisms that regulate *Mmp2*/MMP-2 expression and activity, along with the development of selective inhibitors, will be essential for advancing the therapeutic potential of MMP-2 modulation.

## 7. Conclusions

Matrix metalloproteinase-2 (MMP-2) is a pivotal enzyme in both normal tissue physiology and disease pathogenesis. Its role in ECM degradation, tissue remodeling, and cell migration is critical for processes such as wound healing, angiogenesis as well as embryogenesis. However, its overactivation is implicated in numerous diseases, including cardiovascular disease, diabetic complications, renal function impairment, and numerous other pathologies. It needs to be underlined that MMP-2 regulation is a multifaceted process involving the balance between activation and inhibition, as well as the influence of growth factors, signaling pathways, and environmental factors like hypoxia, inflammation, and oxidative stress. The well-balanced regulation of MMP-2 is critical for maintaining ECM homeostasis and preventing disease progression. Targeting the regulatory mechanisms of MMP-2, particularly in pathological conditions like fibrosis and cancer, holds significant therapeutic potential but requires a detailed understanding of its complex regulatory network.

## Data Availability

No new data were created or analyzed in this study.

## References

[1] Fanjul-Fernández M., Folgueras A.R., Cabrera S., López-Otín C. (2010). Matrix metalloproteinases: Evolution, gene regulation and functional analysis in mouse models. Biochim. Biophys. Acta.

[2] Zitka O., Kukacka J., Krizkova S., Huska D., Adam V., Masarik M., Prusa R., Kizek R. (2010). Matrix metalloproteinases. Curr. Med. Chem..

[3] Wang X., Khalil R.A. (2018). Matrix Metalloproteinases, Vascular Remodeling, and Vascular Disease. Adv. Pharmacol..

[4] Cui N., Hu M., Khalil R.A. (2017). Biochemical and Biological Attributes of Matrix Metalloproteinases. Prog. Mol. Biol. Transl. Sci..

[5] Wang Q., Shao G., Zhao X., Wong H.H., Chin K., Zhao M., Bai A., Bloom M.S., Love Z.Z., Chu C.R. (2024). Dysregulated fibrinolysis and plasmin activation promote the pathogenesis of osteoarthritis. JCI Insight.

[6] Sanyal S., Amin S.A., Banerjee P., Gayen S., Jha T. (2022). A review of MMP-2 structures and binding mode analysis of its inhibitors to strategize structure-based drug design. Bioorg Med. Chem..

[7] Takeuchi T., Hayashi M., Tamita T., Nomura Y., Kojima N., Mitani A., Takeda T., Hitaka K., Kato Y., Kamitani M. (2022). Discovery of Aryloxyphenyl-Heptapeptide Hybrids as Potent and Selective Matrix Metalloproteinase-2 Inhibitors for the Treatment of Idiopathic Pulmonary Fibrosis. J. Med. Chem..

[8] Cui Q., Wang X., Zhang Y., Shen Y., Qian Y. (2023). Macrophage-Derived MMP-9 and MMP-2 are Closely Related to the Rupture of the Fibrous Capsule of Hepatocellular Carcinoma Leading to Tumor Invasion. Biol. Proced. Online.

[9] Kessenbrock K., Wang C.Y., Werb Z. (2015). Matrix metalloproteinases in stem cell regulation and cancer. Matrix Biol..

[10] Chen Y.J., Jeng J.H., Chang H.H., Huang M.Y., Tsai F.F., Yao C.C. (2013). Differential regulation of collagen, lysyl oxidase and MMP-2 in human periodontal ligament cells by low- and high-level mechanical stretching. J. Periodontal Res..

[11] Terni B., Ferrer I. (2015). Abnormal Expression and Distribution of MMP2 at Initial Stages of Alzheimer’s Disease-Related Pathology. J. Alzheimers Dis..

[12] Lian G.Y., Wang Q.M., Mak T.S., Huang X.R., Yu X.Q., Lan H.Y. (2021). Inhibition of tumor invasion and metastasis by targeting TGF-β-Smad-MMP2 pathway with Asiatic acid and Naringenin. Mol. Ther. Oncolytics.

[13] Quintero-Fabián S., Arreola R., Becerril-Villanueva E., Torres-Romero J.C., Arana-Argáez V., Lara-Riegos J., Ramírez-Camacho M.A., Alvarez-Sánchez M.E. (2019). Role of Matrix Metalloproteinases in Angiogenesis and Cancer. Front. Oncol..

[14] Gearing A.J., Beckett P., Christodoulou M., Churchill M., Clements J., Davidson A.H., Drummond A.H., Galloway W.A., Gilbert R., Gordon J.L. (1994). Processing of tumour necrosis factor-alpha precursor by metalloproteinases. Nature.

[15] Waszczykowska A., Podgórski M., Waszczykowski M., Gerlicz-Kowalczuk Z., Jurowski P. (2020). Matrix Metalloproteinases MMP-2 and MMP-9, Their Inhibitors TIMP-1 and TIMP-2, Vascular Endothelial Growth Factor and sVEGFR-2 as Predictive Markers of Ischemic Retinopathy in Patients with Systemic Sclerosis-Case Series Report. Int. J. Mol. Sci..

[16] Takeuchi T., Nomura Y., Tamita T., Nishikawa R., Kakinuma H., Kojima N., Hitaka K., Tamura Y., Kamitani M., Mima M. (2023). Discovery of TP0597850: A Selective, Chemically Stable, and Slow Tight-Binding Matrix Metalloproteinase-2 Inhibitor with a Phenylbenzamide-Pentapeptide Hybrid Scaffold. J. Med. Chem..

[17] Öner Ç., Köser F., Çolak E. (2024). The association of piR-651 and piR-823 on metastatic and invasive characteristics of triple negative breast cancer cells. Nucleosides Nucleotides Nucleic Acids.

[18] Oselusi S.O., Sibuyi N.R., Martin D.R., Meyer M., Madiehe A.M. (2024). Potential matrix metalloproteinase 2 and 9 inhibitors identified from Ehretia species for the treatment of chronic wounds—Computational drug discovery approaches. Comput. Biol. Med..

[19] Azevedo A., Prado A.F., Antonio R.C., Issa J.P., Gerlach R.F. (2014). Matrix metalloproteinases are involved in cardiovascular diseases. Basic. Clin. Pharmacol. Toxicol..

[20] Mata K.M., Prudente P.S., Rocha F.S., Prado C.M., Floriano E.M., Elias J., Rizzi E., Gerlach R.F., Rossi M.A., Ramos S.G. (2011). Combining two potential causes of metalloproteinase secretion causes abdominal aortic aneurysms in rats: A new experimental model. Int. J. Exp. Pathol..

[21] Xiong W., Knispel R., Mactaggart J., Baxter B.T. (2006). Effects of tissue inhibitor of metalloproteinase 2 deficiency on aneurysm formation. J. Vasc. Surg..

[22] Vacek T.P., Rehman S., Neamtu D., Yu S., Givimani S., Tyagi S.C. (2015). Matrix metalloproteinases in atherosclerosis: Role of nitric oxide, hydrogen sulfide, homocysteine, and polymorphisms. Vasc. Health Risk Manag..

[23] Momi S., Falcinelli E., Petito E., Ciarrocca Taranta G., Ossoli A., Gresele P. (2022). Matrix metalloproteinase-2 on activated platelets triggers endothelial PAR-1 initiating atherosclerosis. Eur. Heart J..

[24] Metwally E., Sanchez Solano A., Lavanderos B., Yamasaki E., Thakore P., McClenaghan C., Rios N., Radi R., Feng Earley Y., Nichols C.G. (2024). Mitochondrial Ca2+-coupled generation of reactive oxygen species, peroxynitrite formation, and endothelial dysfunction in Cantú syndrome. JCI Insight.

[25] Souza C.R.R., Caetano E.S.P., Rodrigues S.D., Lopes M.C., Mattos B.R., Santos M.L., Rizzi E., Dias-Junior C.A. (2024). Isoflurane increases the activity of the vascular matrix metalloproteinase-2 in non-pregnant rats and increases the nitric oxide metabolites in pregnancy. Biosci. Rep..

[26] Prado A.F., Batista R.I.M., Tanus-Santos J.E., Gerlach R.F. (2021). Matrix Metalloproteinases and Arterial Hypertension: Role of Oxidative Stress and Nitric Oxide in Vascular Functional and Structural Alterations. Biomolecules.

[27] Olejarz W., Łacheta D., Kubiak-Tomaszewska G. (2020). Matrix Metalloproteinases as Biomarkers of Atherosclerotic Plaque Instability. Int. J. Mol. Sci..

[28] Koudstaal T., Boomars K.A., Kool M. (2020). Pulmonary Arterial Hypertension and Chronic Thromboembolic Pulmonary Hypertension: An Immunological Perspective. J. Clin. Med..

[29] Viswanathan G., Kirshner H.F., Nazo N., Ali S., Ganapathi A., Cumming I., Zhuang Y., Choi I., Warman A., Jassal C. (2023). Single-Cell Analysis Reveals Distinct Immune and Smooth Muscle Cell Populations that Contribute to Chronic Thromboembolic Pulmonary Hypertension. Am. J. Respir. Crit. Care Med..

[30] Zhu M.M., Dai J., Dai Z., Peng Y., Zhao Y.Y. (2024). GCN2 kinase activation mediates pulmonary vascular remodeling and pulmonary arterial hypertension. JCI Insight.

[31] Russell J.J., Grisanti L.A., Brown S.M., Bailey C.A., Bender S.B., Chandrasekar B. (2021). Reversion inducing cysteine rich protein with Kazal motifs and cardiovascular diseases: The RECKlessness of adverse remodeling. Cell Signal.

[32] Matsuzaki T., Keene D.R., Nishimoto E., Noda M. (2021). Reversion-inducing cysteine-rich protein with Kazal motifs and MT1-MMP promote the formation of robust fibrillin fibers. J. Cell Physiol..

[33] Medzikovic L., Aryan L., Ruffenach G., Li M., Savalli N., Sun W., Sarji S., Hong J., Sharma S., Olcese R. (2023). Myocardial fibrosis and calcification are attenuated by microRNA-129-5p targeting Asporin and Sox9 in cardiac fibroblasts. JCI Insight.

[34] Kamiński T.W., Pawlak K., Karbowska M., Myśliwiec M., Pawlak D. (2017). Indoxyl sulfate—The uremic toxin linking hemostatic system disturbances with the prevalence of cardiovascular disease in patients with chronic kidney disease. BMC Nephrol..

[35] Tahir U.A., Kolm P., Kwong R.Y., Desai M.Y., Dolman S.F., Deng S., Appelbaum E., Desvigne-Nickens P., DiMarco J.P., Tiwari G. (2024). Protein Biomarkers of Adverse Clinical Features and Events in Sarcomeric Hypertrophic Cardiomyopathy. Circ. Heart Fail..

[36] Wolosowicz M., Prokopiuk S., Kaminski T.W. (2022). Recent Advances in the Treatment of Insulin Resistance Targeting Molecular and Metabolic Pathways: Fighting a Losing Battle?. Medicina.

[37] Murakami T., Inagaki N., Kondoh H. (2022). Cellular Senescence in Diabetes Mellitus: Distinct Senotherapeutic Strategies for Adipose Tissue and Pancreatic β Cells. Front. Endocrinol..

[38] Arreguin-Cano J.A., Ayerdi-Nájera B., Tacuba-Saavedra A., Navarro-Tito N., Dávalos-Martínez A., Emigdio-Vargas A., Barrera-Rodríguez E., Blanco-García N., Gutiérrez-Venegas G., Ventura-Molina E. (2019). MMP-2 salivary activity in type 2 diabetes mellitus patients. Diabetol. Metab. Syndr..

[39] Ispanovic E., Haas T.L. (2006). JNK and PI3K differentially regulate MMP-2 and MT1-MMP mRNA and protein in response to actin cytoskeleton reorganization in endothelial cells. Am. J. Physiol. Cell Physiol..

[40] Sebastiano M., Momi S., Falcinelli E., Bury L., Hoylaerts M.F., Gresele P. (2017). A novel mechanism regulating human platelet activation by MMP-2-mediated PAR1 biased signaling. Blood.

[41] Ghionescu A.V., Sorop A., Linioudaki E., Coman C., Savu L., Fogarasi M., Lixandru D., Dima S.O. (2024). A predicted epithelial-to-mesenchymal transition-associated mRNA/miRNA axis contributes to the progression of diabetic liver disease. Sci. Rep..

[42] Tsioufis C., Bafakis I., Kasiakogias A., Stefanadis C. (2012). The role of matrix metalloproteinases in diabetes mellitus. Curr. Top. Med. Chem..

[43] Liu J., Xie X., Yan D., Wang Y., Yuan H., Cai Y., Luo J., Xu A., Huang Y., Cheung C.W. (2020). Up-regulation of FoxO1 contributes to adverse vascular remodelling in type 1 diabetic rats. J. Cell Mol. Med..

[44] Li Y., Li L., Zeng O., Liu J.M., Yang J. (2017). H2S improves renal fibrosis in STZ-induced diabetic rats by ameliorating TGF-β1 expression. Ren. Fail..

[45] Shiau M.Y., Tsai S.T., Tsai K.J., Haung M.L., Hsu Y.T., Chang Y.H. (2006). Increased circulatory MMP-2 and MMP-9 levels and activities in patients with type 1 diabetes mellitus. Mt. Sinai J. Med..

[46] Falkowski B., Rogowicz-Frontczak A., Szczepanek-Parulska E., Krygier A., Wrotkowska E., Uruska A., Araszkiewicz A., Ruchala M., Zozulinska-Ziolkiewicz D. (2020). Novel Biochemical Markers of Neurovascular Complications in Type 1 Diabetes Patients. J. Clin. Med..

[47] Yürük Yıldırım Z., Yılmaz A., Pehlivanoğlu C., Gedikbaşı A., Yıldız M., Dirican A., Bundak R., Darendeliler F., Emre S., Nayır A. (2019). Urine Levels of Matrix Metalloproteinases and Tissue Inhibitor of Metalloproteinases in Children with Type 1 Diabetes Mellitus. J. Clin. Res. Pediatr. Endocrinol..

[48] Derosa G., Avanzini M.A., Geroldi D., Fogari R., Lorini R., De Silvestri A., Tinelli C., Rondini G., d’Annunzio G. (2005). Matrix metalloproteinase 2 may be a marker of microangiopathy in children and adolescents with type 1 diabetes mellitus. Diabetes Res. Clin. Pract..

[49] Song H., Sontz R.A., Vance M.J., Morris J.M., Sheriff S., Zhu S., Duan S., Zeng J., Koeppe E., Pandey R. (2023). High-fat diet plus HNF1A variant promotes polyps by activating β-catenin in early-onset colorectal cancer. JCI Insight.

[50] Mansoori A., Nosrati M., Dorchin M., Mohammadyari F., Derakhshan-Nezhad E., Ferns G., Esmaily H., Ghayour-Mobarhan M. (2024). A novel index for diagnosis of type 2 diabetes mellitus: Cholesterol, High density lipoprotein, and Glucose (CHG) index. J. Diabetes Investig..

[51] Shi X., Yang M., Jiang X., Li Y., Meng L. (2023). Correlation of MMP-2, TIMP-1, β2-MG and hs-CRP with the progression of retinopathy in patients with type 2 diabetes. Cell Mol Biol.

[52] Lv J., Cao C.J., Li W., Li S.L., Zheng J., Yang X.L. (2023). Tear inflammation related indexes after cataract surgery in elderly patients with type 2 diabetes mellitus. World J. Clin. Cases.

[53] Xiang Y., Wang Z., Hui Q., Gwinn M., Vaccarino V., Sun Y.V. (2021). DNA Methylation of TXNIP Independently Associated with Inflammation and Diabetes Mellitus in Twins. Twin Res. Hum. Genet..

[54] Preil S.A.R., Thorsen A.F., Christiansen A.L., Poulsen M.K., Karsdal M.A., Leeming D.J., Rasmussen L.M. (2017). Is cardiovascular disease in patients with diabetes associated with serum levels of MMP-2, LOX, and the elastin degradation products ELM and ELM-2?. Scand. J. Clin. Lab. Investig..

[55] Buraczynska M., Dragan M., Buraczynska K., Orlowska-Kowalik G., Ksiazek A. (2015). Matrix metalloproteinase-2 (MMP-2) gene polymorphism and cardiovascular comorbidity in type 2 diabetes patients. J. Diabetes Complicat..

[56] Sarray S., Lamine L.B., Dallel M., Jairajpuri D., Turki A., Sellami N., Ezzidi I., Abdelhadi M., Brock R., Ghorbel M. (2022). Association of MMP-2 genes variants with diabetic retinopathy in Tunisian population with type 2 diabetes. J. Diabetes Complicat..

[57] Kozakova M., Morizzo C., Goncalves I., Natali A., Nilsson J., Palombo C. (2019). Cardiovascular organ damage in type 2 diabetes mellitus: The role of lipids and inflammation. Cardiovasc. Diabetol..

[58] Mercado M.G., Smith D.K., Guard E.L. (2019). Acute Kidney Injury: Diagnosis and Management. Am. Fam. Physician.

[59] Ceron C.S., Baligand C., Joshi S., Wanga S., Cowley P.M., Walker J.P., Song S.H., Mahimkar R., Baker A.J., Raffai R.L. (2017). An intracellular matrix metalloproteinase-2 isoform induces tubular regulated necrosis: Implications for acute kidney injury. Am. J. Physiol. Ren. Physiol..

[60] McNair E.D., Bezaire J., Moser M., Mondal P., Conacher J., Franczak A., Sawicki G., Reid D., Khani-Hanjani A. (2021). The Association of Matrix Metalloproteinases with Acute Kidney Injury Following CPB-Supported Cardiac Surgery. Can. J. Kidney Health Dis..

[61] Kaneko T., Shimizu A., Mii A., Fujita E., Fujino T., Kunugi S., Du X., Akimoto T., Tsuruoka S., Ohashi R. (2012). Role of matrix metalloproteinase-2 in recovery after tubular damage in acute kidney injury in mice. Nephron Exp. Nephrol..

[62] Wang W., Shen Q., Zhou X. (2023). The predictive value of [TIMP-2]*[IGFBP7] in adverse outcomes for acute kidney injury: A systematic review and meta-analysis. Ren. Fail..

[63] Bihorac A., Chawla L.S., Shaw A.D., Al-Khafaji A., Davison D.L., Demuth G.E., Fitzgerald R., Gong M.N., Graham D.D., Gunnerson K. (2014). Validation of cell-cycle arrest biomarkers for acute kidney injury using clinical adjudication. Am. J. Respir. Crit. Care Med..

[64] Sharma A., Kilari S., Cai C., Simeon M.L., Misra S. (2020). Increased fibrotic signaling in a murine model for intra-arterial contrast-induced acute kidney injury. Am. J. Physiol. Ren. Physiol..

[65] Wyczanska M., Rohling J., Keller U., Benz M.R., Kirschning C., Lange-Sperandio B. (2023). TLR2 mediates renal apoptosis in neonatal mice subjected experimentally to obstructive nephropathy. PLoS ONE.

[66] Kaminski T.W., Pawlak K., Karbowska M., Mysliwiec M., Grzegorzewski W., Kuna J., Pawlak D. (2018). Association between uremic toxin-anthranilic acid and fibrinolytic system activity in predialysis patients at different stages of chronic kidney disease. Int. Urol. Nephrol..

[67] Kalantar-Zadeh K., Jafar T.H., Nitsch D., Neuen B.L., Perkovic V. (2021). Chronic kidney disease. Lancet.

[68] Baudier R.L., Orlandi P.F., Yang W., Chen H.Y., Bansal N., Blackston J.W., Chen J., Deo R., Dobre M., He H. (2024). Matrix Metalloproteinase-2 and CKD Progression: The Chronic Renal Insufficiency Cohort (CRIC) Study. Kidney Med..

[69] Cheng Z., Limbu M.H., Wang Z., Liu J., Liu L., Zhang X., Chen P., Liu B. (2017). MMP-2 and 9 in Chronic Kidney Disease. Int. J. Mol. Sci..

[70] Kobusiak-Prokopowicz M., Krzysztofik J., Kaaz K., Jolda-Mydlowska B., Mysiak A. (2018). MMP-2 and TIMP-2 in Patients with Heart Failure and Chronic Kidney Disease. Open Med..

[71] Musiał K., Bargenda A., Zwolińska D. (2015). Urine matrix metalloproteinases and their extracellular inducer EMMPRIN in children with chronic kidney disease. Ren. Fail..

[72] Liao L., Tao P., Xu Q., Chen J., Liu W., Hu J., Lu J. (2024). Bushen Huoxue formula protects against renal fibrosis and pyroptosis in chronic kidney disease by inhibiting ROS/NLRP3-mediated inflammasome activation. Ren. Fail..

[73] Feng X., Zhang J., Yang R., Bai J., Deng B., Cheng L., Gao F., Xie J., Zhang B. (2023). The CaMKII Inhibitory Peptide AIP Alleviates Renal Fibrosis Through the TGF-β/Smad and RAF/ERK Pathways. J. Pharmacol. Exp. Ther..

[74] Takamiya Y., Fukami K., Yamagishi S., Kaida Y., Nakayama Y., Obara N., Iwatani R., Ando R., Koike K., Matsui T. (2013). Experimental diabetic nephropathy is accelerated in matrix metalloproteinase-2 knockout mice. Nephrol. Dial. Transplant..

[75] Młynarczyk G., Gudowska-Sawczuk M., Mroczko B., Bruczko-Goralewska M., Romanowicz L., Tokarzewicz A. (2023). Higher Content but No Specific Activity in Gelatinase B (MMP-9) Compared with Gelatinase A (MMP-2) in Human Renal Carcinoma. Cancers.

[76] Barbe A.M., Berbets A.M., Davydenko I.S., Koval H.D., Yuzko V.O., Yuzko O.M. (2020). Expression and Significance of Matrix Metalloproteinase-2 and Matrix Metalloproteinas-9 in Endometriosis. J. Med. Life..

[77] Deady L.D., Sun J. (2015). A Follicle Rupture Assay Reveals an Essential Role for Follicular Adrenergic Signaling in Drosophila Ovulation. PLoS Genet..

[78] Atabakhsh M., Khodadadi I., Amiri I., Mahjub H., Tavilani H. (2018). Activity of Matrix Metalloproteinase 2 and 9 in Follicular Fluid and Seminal Plasma and Its Relation to Embryo Quality and Fertilization Rate. J. Reprod. Infertil..

[79] Hrabia A. (2021). Matrix Metalloproteinases (MMPs) and Inhibitors of MMPs in the Avian Reproductive System: An Overview. Int. J. Mol. Sci..

[80] Kalev-Altman R., Becker G., Levy T., Penn S., Shpigel N.Y., Monsonego-Ornan E., Sela-Donenfeld D. (2023). Mmp2 Deficiency Leads to Defective Parturition and High Dystocia Rates in Mice. Int. J. Mol. Sci..

[81] Verslegers M., Lemmens K., Van Hove I., Moons L. (2013). Matrix metalloproteinase-2 and -9 as promising benefactors in development, plasticity and repair of the nervous system. Prog. Neurobiol..

[82] Bajor M., Kaczmarek L. (2013). Proteolytic remodeling of the synaptic cell adhesion molecules (CAMs) by metzincins in synaptic plasticity. Neurochem. Res..

[83] Brule S., Charnaux N., Sutton A., Ledoux D., Chaigneau T., Saffar L., Gattegno L. (2006). The shedding of syndecan-4 and syndecan-1 from HeLa cells and human primary macrophages is accelerated by SDF-1/CXCL12 and mediated by the matrix metalloproteinase-9. Glycobiology.

[84] Wang Q., Uhlirova M., Bohmann D. (2010). Spatial restriction of FGF signaling by a matrix metalloprotease controls branching morphogenesis. Dev. Cell.

[85] Rempe R.G., Hartz A.M.S., Bauer B. (2016). Matrix metalloproteinases in the brain and blood-brain barrier: Versatile breakers and makers. J. Cereb. Blood Flow. Metab..

[86] Song J., Wu C., Korpos E., Zhang X., Agrawal S.M., Wang Y., Faber C., Schäfers M., Körner H., Opdenakker G. (2015). Focal MMP-2 and MMP-9 activity at the blood-brain barrier promotes chemokine-induced leukocyte migration. Cell Rep..

[87] Shi T., Yue S., Xie C., Li X., Yang D., Hu L., Zhong Y., Zhang Y., Liu W. (2024). MMP-2-mediated Scube2 degradation promotes blood-brain barrier disruption by blocking the interaction between astrocytes and endothelial cells via inhibiting Sonic hedgehog pathway during early cerebral ischemia. J. Neurochem..

[88] Niland S., Riscanevo A.X., Eble J.A. (2021). Matrix Metalloproteinases Shape the Tumor Microenvironment in Cancer Progression. Int. J. Mol. Sci..

[89] Liu Z.L., Chen H.H., Zheng L.L., Sun L.P., Shi L. (2023). Angiogenic signaling pathways and anti-angiogenic therapy for cancer. Signal Transduct. Target. Ther..

[90] Yu C.F., Chen F.H., Lu M.H., Hong J.H., Chiang C.S. (2017). Dual roles of tumour cells-derived matrix metalloproteinase 2 on brain tumour growth and invasion. Br. J. Cancer.

[91] Su Y., Iacob R.E., Li J., Engen J.R., Springer T.A. (2022). Dynamics of integrin α5β1, fibronectin, and their complex reveal sites of interaction and conformational change. J. Biol. Chem..

[92] Li T., Cao H., Wu S., Zhong P., Ding J., Wang J., Wang F., He Z., Huang G.-L. (2022). Phosphorylated ATF1 at Thr184 promotes metastasis and regulates MMP2 expression in gastric cancer. J. Transl. Med..

[93] Bornschein J., Seidel T., Langner C., Link A., Wex T., Selgrad M., Jechorek D., Meyer F., Bird-Lieberman E., Vieth M. (2015). MMP2 and MMP7 at the invasive front of gastric cancer are not associated with mTOR expression. Diagn. Pathol..

[94] Wang X., Yang B., She Y., Ye Y. (2018). The lncRNA TP73-AS1 promotes ovarian cancer cell proliferation and metastasis via modulation of MMP2 and MMP9. J. Cell Biochem..

[95] Peng K., Zhang Y., Liu D., Chen J. (2023). MMP2 is a immunotherapy related biomarker and correlated with cancer-associated fibroblasts infiltrate in melanoma. Cancer Cell Int..

[96] Zeng W., Su J., Wu L., Yang D., Long T., Li D., Kuang Y., Li J., Qi M., Zhang J. (2014). CD147 promotes melanoma progression through hypoxia-induced MMP2 activation. Curr. Mol. Med..

[97] Nishida Y., Miyamori H., Thompson E.W., Takino T., Endo Y., Sato H. (2008). Activation of matrix metalloproteinase-2 (MMP-2) by membrane type 1 matrix metalloproteinase through an artificial receptor for proMMP-2 generates active MMP-2. Cancer Res..

[98] Paiva K.B.S., Granjeiro J.M. (2017). Matrix Metalloproteinases in Bone Resorption, Remodeling, and Repair. Prog. Mol. Biol. Transl. Sci..

[99] Jiang L., Sheng K., Wang C., Xue D., Pan Z. (2021). The Effect of MMP-2 Inhibitor 1 on Osteogenesis and Angiogenesis During Bone Regeneration. Front. Cell Dev. Biol..

[100] Borkham-Kamphorst E., Alexi P., Tihaa L., Haas U., Weiskirchen R. (2015). Platelet-derived growth factor-D modulates extracellular matrix homeostasis and remodeling through TIMP-1 induction and attenuation of MMP-2 and MMP-9 gelatinase activities. Biochem. Biophys. Res. Commun..

[101] Khoswanto C. (2023). Role of matrix metalloproteinases in bone regeneration: Narrative review. J. Oral. Biol. Craniofac Res..

[102] Reduta T., Bacharewicz-Szczerbicka J., Stasiak-Barmuta A., Kaminski T.W., Flisiak I. (2023). Osteopontin and Regulatory T Cells in Effector Phase of Allergic Contact Dermatitis. J. Clin. Med..

[103] Bauvois B. (2012). New facets of matrix metalloproteinases MMP-2 and MMP-9 as cell surface transducers: Outside-in signaling and relationship to tumor progression. Biochim. Biophys. Acta.

[104] Mohammadhosayni M., Khosrojerdi A., Lorian K., Aslani S., Imani D., Razi B., Babaie F., Torkamandi S. (2020). Matrix metalloproteinases (MMPs) family gene polymorphisms and the risk of multiple sclerosis: Systematic review and meta-analysis. BMC Neurol..

[105] Saremi L., Shahbazi S., Ghaffari M.E., Esmaeili S., Lotfipanah S., Amid R., Kadkhodazadeh M. (2024). The Association of Matrix Metalloproteinase-1, -2, -3, -7, and -13 Gene Polymorphisms With Peri-Implantitis in an Iranian Population: A Case-Control Study. Clin. Exp. Dent. Res..

[106] Usategui-Martín R., Pastor-Idoate S., Chamorro A.J., Fernández I., Fernández-Bueno I., Marcos-Martín M., González-Sarmiento R., Carlos Pastor J. (2019). Meta-analysis of the rs243865 MMP-2 polymorphism and age-related macular degeneration risk. PLoS ONE.

[107] Dofara S.G., Chang S.L., Diorio C. (2020). Gene Polymorphisms and Circulating Levels of MMP-2 and MMP-9: A Review of Their Role in Breast Cancer Risk. Anticancer Res..

[108] Archer C.R., Robinson E.L., Drawnel F.M., Roderick H.L. (2017). Endothelin-1 promotes hypertrophic remodelling of cardiac myocytes by activating sustained signalling and transcription downstream of endothelin type A receptors. Cell Signal.

[109] Shaked I., Foo C., Mächler P., Liu R., Cui Y., Ji X., Broggini T., Kaminski T., Suryakant Jadhav S., Sundd P. (2024). A lone spike in blood glucose can enhance the thrombo-inflammatory response in cortical venules. J. Cereb. Blood Flow. Metab..

[110] Yu X.Y., Sun Q., Zhang Y.M., Zou L., Zhao Y.Y. (2022). TGF-β/Smad Signaling Pathway in Tubulointerstitial Fibrosis. Front. Pharmacol..

[111] Sheng X., Zhang C., Zhao J., Xu J., Zhang P., Ding Q., Zhang J. (2024). Microvascular destabilization and intricated network of the cytokines in diabetic retinopathy: From the perspective of cellular and molecular components. Cell Biosci..

[112] Kowluru R.A., Kanwar M. (2009). Oxidative stress and the development of diabetic retinopathy: Contributory role of matrix metalloproteinase-2. Free Radic. Biol. Med..

[113] Bartosiewicz J., Kaminski T., Pawlak K., Karbowska M., Tankiewicz-Kwedlo A., Pawlak D. (2017). The activation of the kynurenine pathway in a rat model with renovascular hypertension. Exp. Biol. Med..

[114] Znorko B., Oksztulska-Kolanek E., Michałowska M., Kamiński T., Pawlak K. (2017). Does the OPG/RANKL system contribute to the bone-vascular axis in chronic kidney disease? A systematic review. Adv. Med. Sci..

[115] Chetty C., Lakka S.S., Bhoopathi P., Rao J.S. (2010). MMP-2 alters VEGF expression via alphaVbeta3 integrin-mediated PI3K/AKT signaling in A549 lung cancer cells. Int. J. Cancer.

[116] Azevedo Martins J.M., Rabelo-Santos S.H., do Amaral Westin M.C., Zeferino L.C. (2020). Tumoral and stromal expression of MMP-2, MMP-9, MMP-14, TIMP-1, TIMP-2, and VEGF-A in cervical cancer patient survival: A competing risk analysis. BMC Cancer.

[117] https://clinicaltrials.gov/study/NCT05670834.

[118] https://clinicaltrials.gov/study/NCT04773028.

[119] https://clinicaltrials.gov/study/NCT06628479.

